# Experimental climate change impacts on Baltic coastal wetland plant communities

**DOI:** 10.1038/s41598-022-24913-z

**Published:** 2022-11-27

**Authors:** T. F. Bergamo, R. D. Ward, C. B. Joyce, M. Villoslada, K. Sepp

**Affiliations:** 1 Institute of Agriculture and Environmental Sciences, Estonian University of Life Sciences, Kreutzwaldi 13 5, 51014 Tartu, Estonia; 2 Centre for Aquatic Environments, University of Brighton, Cockcroft Building, Moulsecoomb, Brighton, BN2 4GJ UK; 3 Department of Geographical and Historical Studies, University of Eastern Finland, P.O. Box 111, 80101 Joensuu, Finland

**Keywords:** Climate-change ecology, Community ecology, Wetlands ecology

## Abstract

Coastal wetlands provide a range of important ecosystem services, yet they are under threat from a range of stressors including climate change. This is predominantly as a result of alterations to the hydroregime and associated edaphic factors. We used a three-year mesocosm experiment to assess changes in coastal plant community composition for three plant communities in response to altered water level and salinity scenarios. Species richness and abundance were calculated by year and abundance was plotted using rank abundance curves. The permutational multivariate analysis of variance with Bray–Curtis dissimilarity was used to examine differences among treatments in plant community composition. A Non-metric Multi-dimensional Scaling analysis (NMDS) was used to visualize the responses of communities to treatments by year. Results showed that all three plant communities responded differently to altered water levels and salinity. Species richness and abundance increased significantly in an Open Pioneer plant community while Lower and Upper Shore plant communities showed less change. Species abundances changed in all plant communities with shifts in species composition significantly influenced by temporal effects and treatment. The observed responses to experimentally altered conditions highlight the need for conservation of these important ecosystems in the face of predicted climate change, since these habitats are important for wading birds and livestock grazing.

## Introduction

Coastal wetlands are considered valuable ecosystems for their biodiversity and the wide range of ecosystem services they provide, including sediment retention, storm buffering, water filtration, nutrient cycling, flood regulation, carbon sequestration, recreational activities and maintenance of productive coastal fisheries^1–6^. All these ecosystem services contribute positively and directly to human life. However, coastal wetlands worldwide are subject to various impacts resulting from natural and anthropogenic drivers, such as urbanization and residential developments, conversion to agricultural land, as well as sea level rise and related impacts of inundation and erosion^7,8^ due to climate change.

Climate change has been highlighted as one of the main risks to coastal wetlands, especially in low-lying areas^9,10^. As global temperatures increase and warm the oceans, melting ice sheets and glaciers are expected to accelerate the rate of sea level rise^11–13^ and can modify sea water salinity, alongside changes in the amount and distribution of precipitation and changes in wind speed^13–15^. The impacts of climate change vary both geographically and seasonally. For instance, projections for climate change highlight the largest increase in mean temperature in high latitudes of the northern hemisphere and particularly strong increases in heavy precipitation in the tropics as well as in high latitudes^16^.

The consequences of climate change over recent decades are evident in the Baltic Sea region, with modifications in sea water circulation, temperature and salinity^17,18^. A warmer climate results in modifications to precipitation patterns affecting runoff to the Baltic Sea. Seasonally, summer river flows are likely to either decrease or stay the same, while winter flows are predicted to increase^11^. As a result of this, the average salinity of the Baltic Sea is projected to decrease, with the greatest reductions predicted to be in the surface waters of the Danish straits and lowest in the Gulf of Bothnia, while the water levels are predicted to increase^11^. These changes are likely to have an impact on both plant and animal assemblages^19^.

In the Baltic Sea, both water levels and salinity have a strong impact on species distribution and therefore on the structure and composition of aquatic and coastal floral and faunal communities^19,20^. It has been reported that decreases in salinity during the 1980s altered zooplankton species composition favouring freshwater species^21^ and both salinity and water levels have been shown to have a strong influence on coastal plant community composition^20,22^. The alterations to higher water levels and lower salinity may facilitate the settlement of local freshwater or invasive non-native species that can adapt to lower salinities, affecting ecosystem functioning in species-rich Baltic coastal wetlands^11^.

As a result of water level and salinity changes, plant communities in coastal wetlands are expected to be sensitive to climate change^23,24^. Coastal wetland communities often comprise a mosaic of vegetation patches and are classified by similarities in species composition and indicator species^25^. In temperate zones, coastal wetland plant communities are affected by microtopography^26^, soil water condition^27^, soil salinity^28^ as well as management activities^25^.

In order to assess the response of coastal vegetation to altered environmental conditions, mesocosms have previously been used to investigate vegetation richness^29^, seedling establishment^30^ and nutrient transformation capacity by wetlands^31^. Mesocosm experiments enable the manipulation of specific conditions and species^32^ and allow community-level responses to be evaluated by adding greater complexity at larger scales^33^. Therefore, mesocosm experiments constitute a useful tool to assess the potential impacts of climate change on coastal wetland plant communities.

Due to the high importance of coastal wetlands and the impacts of climate change on this ecosystem, it is important to determine how future conditions will influence coastal plant community functioning. Previous studies in the Baltic region assessed changes to coastal wetland plant communities related to: microtopography (^24^, using ecological assessment); management and grazing (^22^, ecological assessment); climate driven changes to precipitation and sea level rise (^24,34^, modelling). However, there have yet to be any studies directly measuring the influence of climate change impacts on plant community composition in Baltic coastal wetlands. The aim of this study was to examine the effects of altered water level and salinity conditions on three different coastal wetland plant communities using medium-term (3-year) mesocosm experiments. The objectives were:

(i) To evaluate changes in species richness and abundance over time.
(ii) To evaluate changes in the plant communities’ composition under different treatments;

## Results

### Species richness and abundance

**Figure 1:**
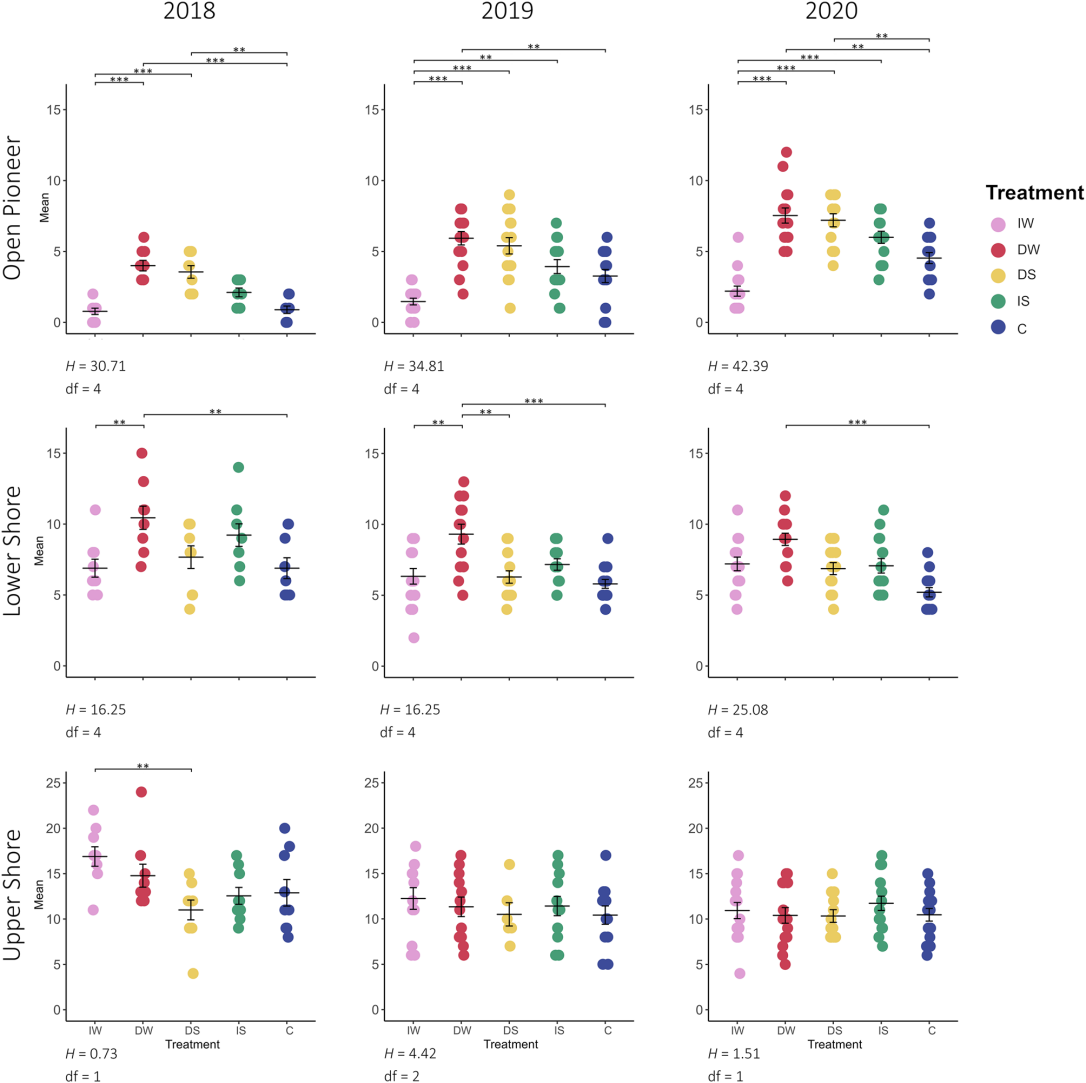
Number of species (species richness) per treatment in 2018, 2019 and 2020 for Open Pioneer, Lower Shore and Upper Shore communities. One dot represents an individual species. The cross represents the standard error (se) (vertical) and mean (horizontal), while horizontal bars indicate significant differences among treatments. The level of significance is coded as **p = 0.01, and ***p = 0.001. Differences in species richness between treatments were examined using Kruskal Wallis followed by Dunn’s post hoc test with Bonferroni adjustments in the years 2018, 2019 and 2020. Kruskal Wallis results are shown as H-value and degrees of freedom (df). Pink: increased water level (IW); red: decreased water level (DW); yellow: decreased salinity (DS); green: increased salinity (IS); blue: control (C). See Table 2 for treatment details. More information can be found in the Supplementary Table S1, Supplementary Figs. S4, S5, and S6.

In total, 16 plant species were recorded in the Open Pioneer (average of 1.2 per 0.25 m^2^), 26 in the Lower Shore (average of 1.8 per 0.25 m^2^) and 48 in the Upper Shore community (average of 3.3 per 0.25 m^2^). Comparing the beginning to the end of the experiment, species richness increased in the Open Pioneer community in all treatments, while in the Lower and Upper Shore communities, the species richness decreased particularly with decreased water level (Fig. 1).

The pairwise comparisons using Dunn's test with Bonferroni adjustments indicated that the increased water level treatment (IW) and decreased salinity (DS) presented significant richness differences (p < 0.05) compared to decreased water level (DW) and control treatments (C) in the Open Pioneer community in 2018. The following year, in addition to the previous differences, treatment IW was significantly different (p < 0.05) from the higher salinity condition (IS). In 2020, no other differences were observed. The Lower Shore community showed significant differences (p < 0.05) between treatments with altered water level in 2018 and 2019 and between lower water level and lower salinity in 2019. In 2020, significant differences (p < 0.05) were present between lower water level and control treatments. The post hoc test revealed significant differences (p < 0.05) between IW and DS in the Upper Shore community in 2018 and no additional significant values (Fig. 1).

**Figure 2:**
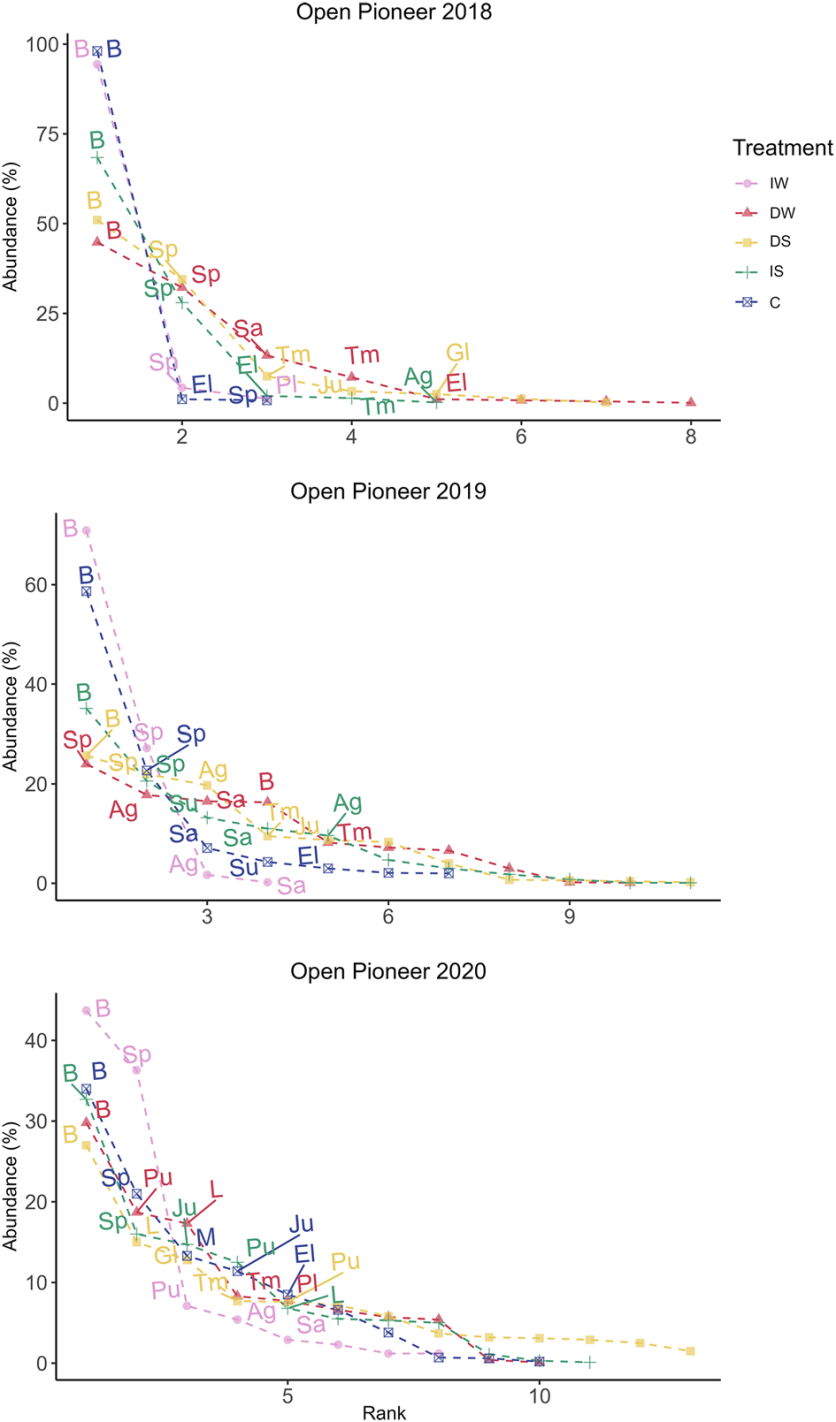
Rank abundance curve of the Open Pioneer community. Mean abundance as percentage cover (n = 3) for August is on the y-axis, while species are ranked consecutively on the x-axis. *B* Bare ground, Sp *Spergularia salina*, Sa *Salicornia europea*, Tm *Triglochin maritima*, El *Eleocharis palustris*, Ju *Juncus gerardii*, Pl *Plantago maritima*, Gl *Glaux maritima*, Ag* Agrostis stolonifera*, Su *Suaeda maritima*, L *Litter*; Pu *Puccinellia maritima*. Pink: increased water level (IW); red: decreased water level (DW); yellow: decreased salinity (DS); green: increased salinity (IS); blue: control (C).

Species-rank abundance curves were calculated for August by year and revealed a few dominating species in all three communities. All species-rank abundance curves show a long tail containing the majority of species contributing to species richness within each community. The Open Pioneer community was characterized by a large proportion of bare ground (Fig. 2) and a few species adapted to relatively high salinity. The rank abundance curves showed that bare ground was the predominant characteristic during the 3 years of the experiment. *Spergularia marina* was the most abundant species in 2018 and 2019 (Fig. 2) for all treatments, except in the Control where *Eleocharis palustris* was the most abundant in 2018. In 2020, the most abundant species was *Puccinellia maritima* in DW (decreased water level) and *Glaux maritima* in DS (decreased salinity) (Fig. 2).

**Figure 3:**
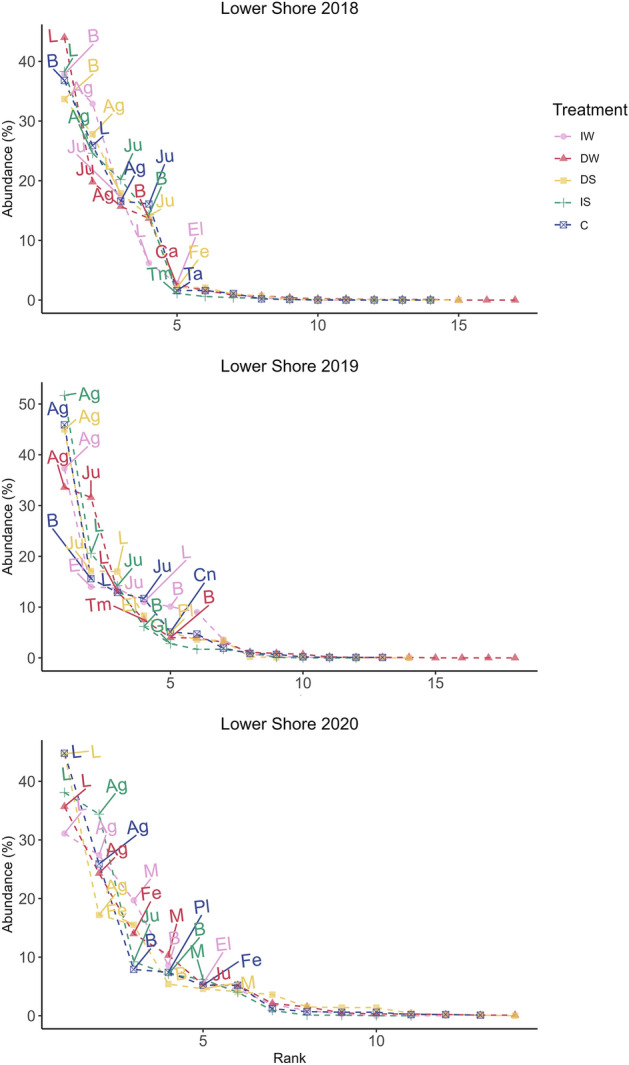
Rank abundance curve of the Lower Shore community. Mean abundance as percentage cover (n = 3) for August is on the y-axis, while species are ranked consecutively on the x-axis. *B* Bare gound, Ag *Agrostis stolonifera*, Ju *Juncus gerardii*, El *Eleocharis palustris*, Pl *Plantago maritima*, Fe *Festuca rubra*, Tm *Triglochin maritima*, *L* Litter; Ca *Carex nigra*, Gl *Glaux maritima*, *M* Moss, Cn *Cnidium dubium*. Pink: increased water level (IW); red: decreased water level (DW); yellow: decreased salinity (DS); green: increased salinity (IS); blue: control (C).

The most abundant species in Lower Shore (Fig. 3) were *Agrostis stolonifera* followed by *Juncus gerardii* in 2018 in all treatments, except in the DW treatment where *Juncus gerardii* was the most abundant followed by *Agrostis stolonifera*. In 2019, *Agrostis stolonifera* was the most abundant species in all treatments, followed by *Eleocharis palustris* in IW (increased water level), and *Juncus gerardii* in the other treatments (DW- decreased water level, DS- decreased salinity, IS- increased salinity, and C- Control) (Fig. 3). Litter was the most abundant attribute in 2020. Considering the species, *Agrostis stolonifera* was the most abundant species, followed by *Festuca rubra* in DW and DS, and *Juncus gerardii* in IS. Generally, the amount of litter increased from the beginning of the experiment to the end, reducing bare ground cover.

**Figure 4:**
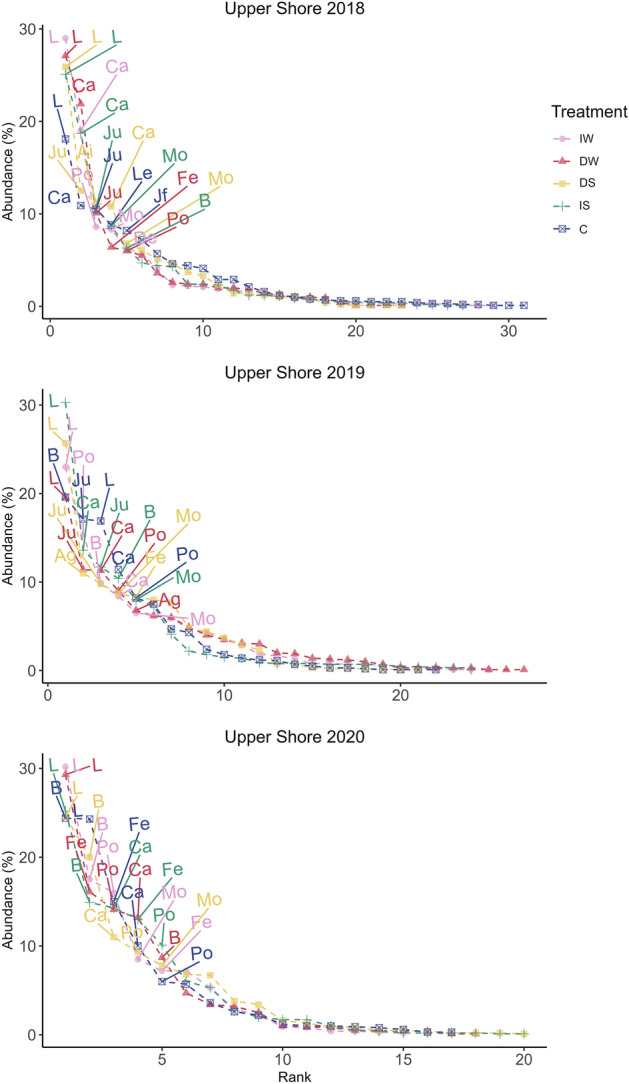
Rank abundance curve of the Upper Shore community. Mean abundance as percentage cover (n = 3) for August is on the y-axis, while species are ranked consecutively on the x-axis. Ca *Carex nigra*, Ju *Juncus gerardii*, Po* Poa annua*, *B* bare ground, *Mo* *Molinea caerulea*, *Le* *Leontodon autumnalis*, Ai *Agrostis gigantea*, De *Deschampsia cespitosa*, Ag *Agrostis stolonifera*, Fe *Festuca rubra*. Pink: increased water level (IW); red: decreased water level (DW); yellow: decreased salinity (DS); green: increased salinity (IS); blue: control (C).

For the Upper Shore community (Fig. 4), litter was most abundant, except in 2018 and 2019. *Carex nigra* was also the most common species in 2018 except in DS where *Juncus gerardii* had a higher abundance. In 2019, *Carex nigra* was the most abundant species in IS, *A. stolonifera* in DS, *Juncus gerardii* in DW and C, and *Poa angustifolia* in IW. In 2020, *Festuca rubra* had a higher abundance in DW and C (decreased water level and control), *Poa angustifolia* in the increased water level treatment (IW), and *Carex nigra* in DS and IS.

### Plant community composition

Different factors (year and treatment) significantly influenced plant community composition. Based on factor effects, year explained most of the variation for Open Pioneer. Year explained the most variation followed by Treatment for the Lower Shore and Upper Shore communities (Table 1).

**Table 1:** PERMANOVA results based on Bray–Curtis dissimilarity from species importance values obtained from the mean percentage cover (n = 3).

Source of variation	Df	MeanSqs	F. model	R^2^	*p* (perm)
**Open Pioneer**					
Treatment	1	0.051	0.56	0.010	
Year	1	1.055	11.627	0.214	< 0.001
Treatment × year	1	0.107	1.1809	0.021	
Residuals	51	3.719		0.754	
Total	41			1	
**Lower Shore**					
Treatment	1	0.191	3.846	0.070	< 0.001
Year	1	0.399	8.019	0.147	< 0.001
Treatment vs year	1	0.779	1.559	0.028	
Residuals	41	2.0429		0.753	
Total	44			1	
**Upper Shore**					
Treatment	1	0.268	3.229	0.063	< 0.001
Year	1	0.486	5.856	0.114	< 0.001
Treatment vs year	1	0.086	1.038	0.020	
Residuals	41	3.406		0.802	
Total	44			1	

The results show the effects of experimental factors on plant community composition (August of 2018, 2019 and 2020).

**Figure 5:**
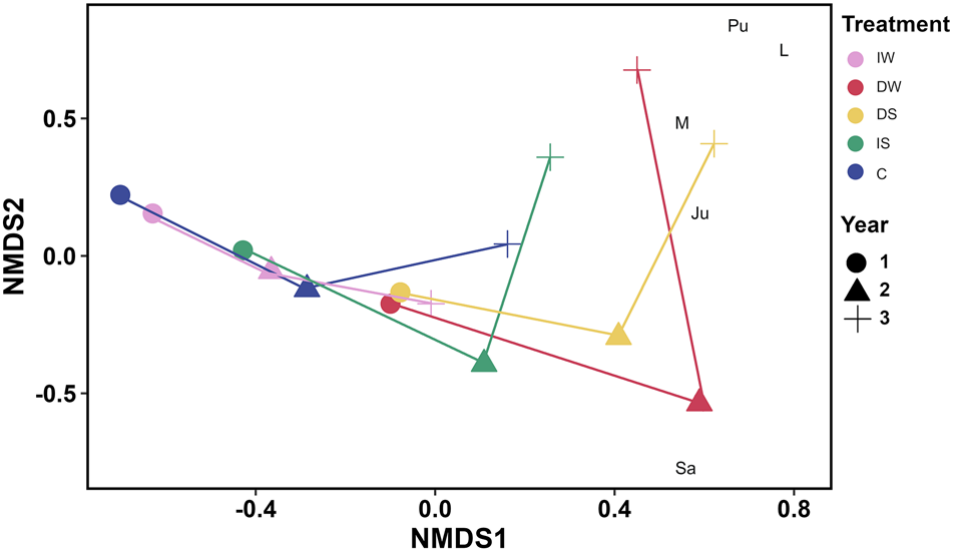
Non-metric multi-dimensional scaling (NMDS) plot of Open Pioneer plant community composition (averaged % cover from August; n = 3) based on Bray–Curtis dissimilarity matrix. Ju *Juncus gerardii*, Sa *Salicornia europaea*, Pu *Puccinellia maritima*, *L* litter, *M* moss. Pink: increased water level (IW); red: decreased water level (DW); yellow: decreased salinity (DS); green: increased salinity (IS); blue: control (C). Circle: year 1 (2018); triangle: year 2 (2019); cross: year 3 (2020).

**Figure 6:**
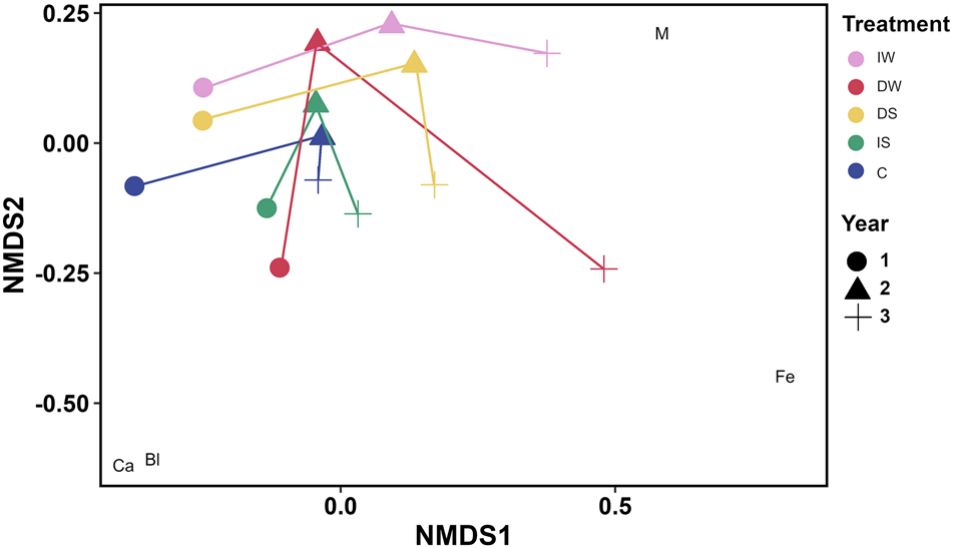
Non-metric multi-dimensional scaling (NMDS) plot of Lower Shore plant community composition (averaged % cover from August; n = 3) based on Bray–Curtis dissimilarity matrix. *M* moss; Fe *Festuca rubra*, Bl *Blysmus rufus*, Ca *Carex nigra*. Pink: increased water level (IW); red: decreased water level (DW); yellow: decreased salinity (DS); green: increased salinity (IS); blue: control (C). Circle: year 1 (2018); triangle: year 2 (2019); cross: year 3 (2020).

**Figure 7:**
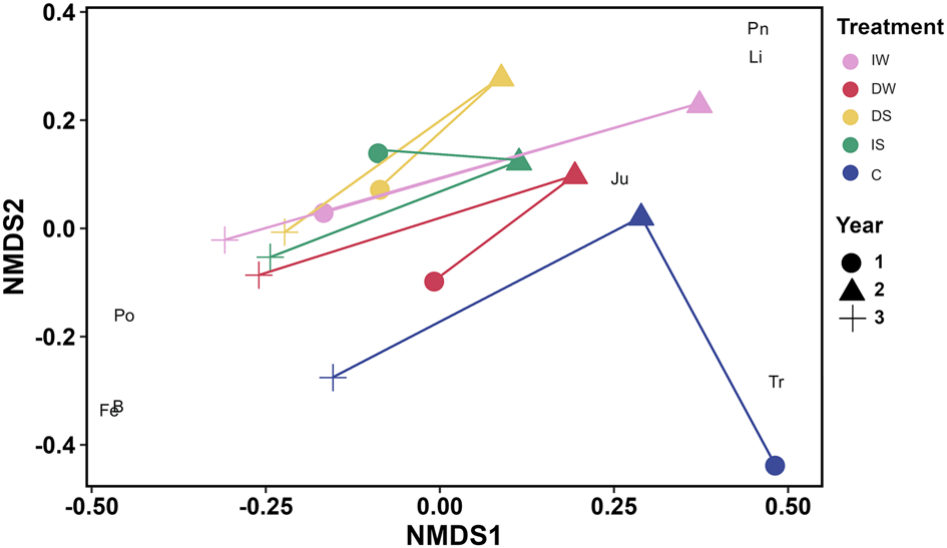
Non-metric multi-dimensional scaling (NMDS) plot of Upper Shore plant community composition averaged % cover from August; n = 3) based on Bray–Curtis dissimilarity matrix. Po *Poa annua*, Pn *Poa angustifolia*, Li *Linum catharticum*, *B* bare ground, Fe *Festuca rubra*, Tr *Triglochin maritima*, Ju *Juncus gerardii*. Pink: increased water level (IW); red: decreased water level (DW); yellow: decreased salinity (DS); green: increased salinity (IS); blue: control (C). Circle: year 1 (2018); triangle: year 2 (2019); cross: year 3 (2020).

The results of Non-Metric Multidimensional scaling (NMDS) indicates the species trajectory during the experiment (measured in August) (Figs. 5, 6 and 7).

The number of species increased over time in the Open Pioneer community. The few indicator species in the community can be associated with salinity changes, such as moss and *Juncus gerardii* indicating decreased salinity conditions. The few species associated with altered conditions in the Lower Shore community were related to increased water level (moss) and decreased water level (*Festuca rubra*).

The Upper Shore community showed species such as *Linum catharticum* and *Poa angustifolia* indicating increased water level and decreased salinity, *Triglochin maritima* related to the control conditions, and *Festuca rubra* to the control at the end of the experiment.

## Discussion

### Changes in coastal wetland composition and abundance

The results demonstrated that all three Baltic coastal wetland communities exhibited considerable temporal changes under altered water and salinity regimes, highlighting the response of plant species to environmental variables. Plant communities and species are generally excellent indicators of environmental conditions^22^. The status of wetland management, disturbance or abandonment have been assessed by plant community composition^25,32^ as they are influenced by biotic (e.g. competition, facilitation and grazing) and abiotic factors (e.g. flooding, salinity and soil nutrients)^23,35,36^. The mesocosm experiment was kept outdoors during the study. For this reason, it was subject to changing weather and environmental conditions. The controls were therefore expected to change over time, providing a reference against which the treatment effects could be compared, since the environmental conditions were the same for all treatments. Despite this observation, plants species have shown to be sensitive indicators to relatively small changes in water levels and salinity.

In coastal wetlands, hydrology and salinity are key variables that generate stress gradients for plants and consequently determine their distribution^22^. Shifts in plant communities were observed over a period of five years in an intertidal saltmarsh in Australia to simulated sea level rise acceleration beyond accretion rates^37^. On the contrary, the present work revealed a rapid change in plant community composition within two years of altered water levels and salinity. These findings were consistent with previous studies following changes in hydrological conditions^38^ and salinity^39^ in wet grasslands.

The Open Pioneer (OP) community showed an increase in species abundance and vegetation cover through the years, while Lower Shore (LS) and Upper Shore (US) underwent changes to a lesser degree (Fig. 1), related to both water level and salinity.

In coastal wetlands, the Open Pioneer community is characterized as patches with distinctive edaphic characteristics compared to surrounding plant communities, with a high abundance of bare ground, high levels of soil conductivity and pH, and low soil moisture^25^. This community presents elevated levels of evaporation and consequently high salinity concentrations. It has a low abundance of vegetation cover, with few characteristic species able to germinate and grow in such conditions (halophytes). Alterations in this community could be observed through the years.

The Open Pioneer community was a variable and dynamic community, responding quickly to the new conditions of water level and salinity. That could explain the significant change over the years and the non-significant effect of treatments on the community. These shifts could have more pronounced alterations and loss of rare species by altered water level and salinity because rising sea levels could increase inundation in previously rarely inundated areas^25^. Species related to conditions of decreased water were observed in the related treatment, such as *Juncus gerardii* (DW). The seed bank in Open Pioneer could explain the rapid colonization of species related to altered conditions, since all the species that established during the experiment are species found in Estonian coastal wetlands and adjacent communities. In this regard, the seed bank often reflects adjacent community composition^40^ and follows site-specific physical conditions^41^. Jutila^42^ found that the total vegetation cover in the Baltic coastal wetlands was positively correlated with seedling density and negatively with open spaces of bare soil. On the other hand, this study revealed emergence and an increase in the abundance of pioneer species within the Open Pioneer, including species that grow in open communities such as *Puccinellia maritima* and *Triglochin maritima.* These two species appeared in treatments characterized by lower water table and lower salinity levels. In the third year of experiment, *Glaux maritima* was the most abundant species in the lowered salinity treatment in Open Pioneer. Ellenberg^43^ classified this species as present in the upper saltmarsh and in brackish conditions. In the elevated salinity concentration in the Open Pioneer community, stress- tolerant species such as halophytes, are able to survive thanks to adaptations like osmotic adjustments, and antioxidant production^44^. An association of *Puccinellia maritima* to decreased salinity and water level was observed in this community. This pattern may be related to the fact that this species are pioneers and dominate disturbed environments^45^.

Lower Shore and Upper Shore communities are characterised by perennial species and are subject to disturbance regimes like herbivory, common in secondary grasslands where grazing is the main management strategy. Besides that, these communities show more stability compared to the Open Pioneer community, and this could be explained by the presence of perennial species and higher plant cover compared to the Open Pioneer community. Also, the indicator species of the Lower Shore and Upper Shore communities are tolerant to changes in water level and salinity^46^.

In the third year*, Agrostis stolonifera* was the most abundant species under all treatments in the Lower Shore community, followed by *Festuca rubra* and *Juncus gerardii* in the increased salinity treatment. Although *Festuca rubra* and *Juncus gerardii* species can occur in saline and non-saline environments, *Juncus gerardii* is noted as having a higher tolerance for saline soils than *Festuca rubra*^43^. On the other hand, *Juncus gerardii* cover decreased under different conditions of water level (DW and IW) and decreased salinity (DS). This species is an indicator of the Lower Shore community^25^ and it has shown to be an important species for livestock grazing on the coastal wetlands due to its nutrition and digestibility^47^.

Species abundance varied between treatments in the Upper Shore community, however *Festuca rubra* was the most abundant in conditions of decreased water level and the Control. This species is one of the indicator species of the community^25^. Ellenberg indicator values^43^ show that *Poa angustifolia* is a moist-site indicator and this species was observed under conditions of increased water level. *Carex nigra* was the most abundant species in conditions of increased salinity (in 2018 and 2019) and decreased salinity (2019). Ellenberg^43^ reported this species as absent from saline sites, and if in coastal situations, only accidental and non-persistent if subjected to saline spray or water. In 2020, the second most abundant species in conditions of increased salinity was *Festuca rubra*, which has more tolerance for saline soils compared to *Carex nigra*^43^.

Coastal wetland species are adapted to saline/brackish water conditions and water level fluctuations^24^. Nevertheless, accelerated sea-level rise can be a risk to coastal wetlands at different scales^48^ resulting in total loss where sea-level rise (including land subsidence/uplift) is greater than net elevation gain (compaction, root growth and sediment deposition)^49^. Salinity alterations can also affect photosynthetic rate and leaf growth, causing necrosis and mortality^50^ depending on species adaptation. This could explain the increase in litter and bare ground in the Lower Shore and Upper Shore communities observed within the water level and soil salinity treatments in this experiment.

The findings show that flooding and salinity had a significant role in the number of species and their relative abundance in coastal plant communities^39^. In addition, the alteration of the coastal wetland plant community composition could affect habitat quality for migrating and breeding birds. Wader species use Estonian coastal wetlands as breeding and feeding grounds, and it has been demonstrated that large (≥ 100 ha) and wide (mean width ≥ 200 m) grazed meadows, with a high water-table and no woody vegetation, provide favourable breeding conditions for waders of international conservation concern^51^.

The predictions for sea level rise are different along the Estonian coast due to geographic variations in isostatic uplift and sediment accretion^3^. Coastal wetlands have been shown to be able to adapt to sea level rise if sediment accumulation rates are high enough^24,52^. However, the rate of accretion varies according to sediment supply^53^, which is related to a range of factors including catchment soil moisture, catchment development and topography, ground surface heat budget, rainfall patterns, river regulation, dredging, vegetation type and growth rate, and long-term climate anomalies^37^. Besides that, coastal wetland vegetation may also be threatened or constrained in the longer term as these plant communities may not be able to migrate further inland with rising sea levels if geomorphic constraints, forests or human infrastructure act as barriers^54^.

The experimental conditions in this study considered isostatic uplift and sediment accretion along the Estonian coast, based on Ward’s^3^ calculations, and the results demonstrated significant species and community changes over 3 years.

### Changes in species richness

Modifications in soil salinity concentrations due to climate change can alter soil redox potential and sulphide concentration leading to species composition shifts^29^. The Open Pioneer community is controlled mostly by salinity concentrations, even within soil composed of greater proportions of coarse, medium and fine sand (as is found in this plant community compared to other adjacent communities), which retain less nutrients. The seed bank allowed the seeds to germinate under simulated salinity changes in the Open Pioneer community. *Spergularia marina* and *Glaux maritima* contributed to increased species richness under conditions of raised and decreased soil salinity concentration, respectively. Hulisz^55^ demonstrated an inverse distribution through the Baltic coast, where *Spergularia marina* together with other halophytes was closer to the waterline in soils with a higher salinity, and *Glaux maritima* was associated with other species with lower soil salinity.

Overall, the Lower Shore and Upper Shore community types did not show significant changes under altered salinity conditions compared to the control treatment. These communities present species occurring in both saline and non-saline situations^43^.

In order to assess changes in plant community composition linked to sea level rise, several studies have previously assessed and simulated the responses of plant communities and species under these conditions^29^. For perennial species, Sharpe & Baldwin^29^ found no influence of flooding conditions on species richness. On the other hand, Gough and Grace^56^ observed a reduction in the number of species number with increased flooding stress, while alleviating flooding did not have an effect on the species richness. The Open Pioneer community underwent an increase in species richness with raised water levels compared to the control. Species adapted to higher water levels such as *Spergularia marina* were in greater abundance in year 3 in the increased water level treatment.

Differences were shown in the Lower Shore in terms of species richness in decreased water levels compared to the control. Species variation over time was related to species with low coverage and wet-site indicators (e.g. *Triglochin palustris*)^43^. The Lower Shore is located in a gradient of water level between Open Pioneer and Upper Shore and this could explain species tolerating a wide range of soil moisture conditions.

The Upper Shore community experienced a loss in the number of species and consequently a decrease in species richness within the increased water level treatment; these were species with low coverage and with low water level requirement (e.g. *Stellaria graminea* and *Viola canina*).

The location of the mesocosm may have influenced the changes observed in the control plots during the experiment, as they were subject to the local environmental conditions, although these were similar to those of the original coastal wetland system. Ideally, an in-situ experimental setting would be able to account more precisely for the environmental conditions in the coastal wetlands under study. However, this setting would considerably increase the costs and workload due to the remote location of the coastal wetlands. Regardless of this constraint, the mesocosm experiment presented here provides valuable insights into shifts in plant communities in Estonian coastal wetlands under future conditions of climate change.

## Methods

### Study site

The Baltic Sea is one of the largest brackish water bodies in the world^57^ due to its relative isolation as a consequence of the narrow connection with the Atlantic Ocean through the Danish Straits^58^. It is strongly influenced by large-scale atmospheric circulation, hydrological processes (e.g. currents and internal mixing) and restricted water exchange in its entrance^11,59^. Salinity within the Baltic Sea is maintained by a pattern of stratification with low salinity surface waters during the spring and early summer, and high salinity bottom waters during the summer^60^. The outflow of low salinity water in the surface and the inflow of higher salinity water at depth maintains the upper layer salinity at about 6–8 psu (practical salinity units) around Estonia and a more saline deep-water layer with about 10–14 psu^59^, although this varies geographically.

Coastal wetlands in Estonia have a very low tidal range (~ 0.02 m), and inundation is predominantly driven by atmospheric pressure and fluctuating meteorological conditions across the North Atlantic and Fennoscandia^58^. As a result, the rate and magnitude of inundation is irregular and varies throughout the coastal landscape^13^. Recent estimates of relative sea level rise from three tide gauges along the Estonian coast are: 1.5–1.7 mm year^−1^ at Tallinn, 1.7–2.1 mm year^−1^ from Narva-Jõesuu and 2.3–2.7 mm year^−1^ at Pärnu^3^.

Along the Baltic Sea coastline, both water levels and salinity have a strong influence on species distribution and therefore on the structure and composition of aquatic and coastal floral and faunal communities^19^. Estonian wetlands cover around 25% of the territory and less than 1% comprises coastal wetlands, including grasslands, reed swamps and salt marshes^34^. The average number of vascular plants in the most diverse Estonian coastal wetlands, such as meadows, varies between 15.2 and 26.1 m^−2^^61^. Estonian coastal wetlands are semi-natural grasslands maintained by moderate human activities, such as livestock grazing or haymaking. Grazing and mowing reduce biomass and therefore the dominance of competitive species^22,62,63^. As a consequence of biomass removal, light competition in the sward is reduced, favouring the coexistence of a high number of species and leading to a greater landscape heterogeneity^64^. They support considerable biodiversity, including rare plant species, breeding and migratory birds^13,22^, influenced by topography and hydrology, and favoured by low grazing intensity^22,25^.

### Experimental design

Six coastal wetland plant communities (Open Pioneer, Club-rush swamp, Reed Swamp, Lower Shore grassland, Upper Shore grassland and Tall Grass) were identified based on a phytosociological classification developed by Burnside et al.^25^. This classification was developed for the region where the mesocosm communities were collected and has proven useful to detect changes in plant community composition due to abandonment and management^65,66^. Three coastal wetland communities were selected for the mesocosm experiment: Lower Shore, Upper Shore and Open Pioneer. These communities were selected for the narrow autecological preference of the key species such as water table level soil and salinity. The Open Pioneer community occupies small patches located in depressions and along water courses, where water evaporates in standing pools forming salt deposits at the surface. The Open Pioneer community is characterised by an abundance of bare ground and high salinity and presence of typical halophyte species such as *Salicornia europaea* and *Suaeda maritima*, considered rare for the region. The Lower Shore and Upper Shore communities are located in specific elevations and sometimes co-exist as a mosaic. The Lower Shore community is found in elevations around 30 cm and the Upper Shore community around 38 cm above mean sea level (msl) and further away from the sea^24,25^. The Lower Shore community is mainly indicated by *Juncus gerardii* with frequent *Festuca rubra, Glaux maritima, Plantago maritima* and *Triglochin maritima*. The Upper Shore has denser vegetation than the Lower Shore and is characterized by the dominant presence of *Festuca rubra*, as well as *Juncus gerardii*, *Leontodon autumnalis*, *Triglochin maritima* and *Plantago maritima*^25^. See Supplementary Fig. S3 for more information.

Fifteen turves measuring 50 × 70 cm and 30 cm deep were collected from each of the three selected plant communities (45 in total) within the Tahu North coastal wetland, northwest Estonia (58^o^58′57.8′′ N, 23^o^34′03.9′′ E). These were transplanted into 90L containers (dimensions 56 × 79 × 32 cm) and transported to Tartu, Estonia. The containers were placed at Eerika experimental station (Institute of Agricultural and Environmental Sciences—Estonian University of Life Sciences). The ground was covered with a fabric to prevent the colonization of grasses around the containers, and all the identified grasses not present in the original site were pulled out during the experiment. The containers were filled with a 2:1:1 soil mixture consisting of commercially washed sharp sand, loam and compost^67^, very similar to the deep substrate of the wetland. A rotating mixer was used to homogenize the base soil.

Each experimental treatment contained three replicate containers for each plant community. A control treatment was maintained with current Eastern Baltic Sea salinity (6.5 psu)^11^ and current average water table level during the growing season below the soil surface for each community (Open Pioneer: 0.1 m; Lower Shore: 0.15 m; Upper Shore: 0.2 m)^22,26^. The treatments simulating climate change included two different salinities used in the Lower Shore and Upper Shore communities: 2.9 psu, the decreased predicted salinity by 2100 in the eastern Baltic^11^, and 13 psu to evaluate the effect of an increase in salinity in the event that there is greater mixing of deeper water (salinity of deep sea water in the Eastern Baltic is about 10–14 psu) and surface water as a result of increased storminess linked to climate change (Table 2). Considering the higher salinity characterising the Open Pioneer community (12.5 psu), the simulation was different for this community (with treatments of 5.6 and 25 psu). Salinity was controlled weekly by adding sea salt or fresh water^68^.

**Table 2:** Treatments in the mesocosm experiment. Salinity is given as practical salinity unit (psu) and water level in relation to the soil surface (m). Water level below control level is denoted with minus and water level above control level is denoted with plus sign. *C* control, *IW* increased water level, *DW* decreased water level, *DS* decreased salinity, *IS* increased salinity.

Treatment	C	IW	DW	DS	IS
Salinity (psu)	6.5 12.5*	6.5 12.5*	6.5 12.5*	2.9 5.6*	13 25*
Water level (m)	Open Pioneer: 0.1 Lower Shore: 0.15 Upper Shore: 0.2	+ 0.12	− 0.12	Control	Control

^(*)^ Refers to Open Pioneer salinity.

Three different water level scenarios were simulated on the basis of three parameters, namely: Global sea level rise rates of 3.3 mm year^−1^^69^, isostatic uplift (2.8 mm year^−1^ on the north coast of Estonia and 0 mm year^−1^ in the south west) and sediment accretion rates (1.9 mm year^−1^ best case scenario)^24^. Resulting from the combination of the abovementioned factors, the future (2100) water level change scenarios were: 0.12 m below current plant community water levels (best case scenario with continued progradation) and 0.12 m above current water level (worst case scenario with inundation), plus a control (current water levels). Taps were placed in the containers at the corresponding water level and the treatments were kept constant during the growing period to simulate the limited water level variation (More information can be found in the Supplementary Figs. S1 and S2).

Over three years of the experiment, plant community responses were evaluated once a month using a 50 cm^2^ permanent graduated quadrat (10 cm^2^ sub-quadrats) during the growing period (April to September). Changes in the abundance of plant species present by area of ground cover were assessed^70^ by the presence and percentage cover within 25 sub-quadrats per sample considering bare ground, moss and litter. All the data was averaged within samples.

Each year of the experiment, grazing was simulated by cutting the vegetation to 10 cm height two weeks after turf greening^22^.

### Statistical analysis

Plant communities were characterized using species richness and percentage cover within individual sub quadrats over three years. For the analysis, all the 25 sub-quadrats per sample (50 cm^2^) were averaged. In order to analyse plant community data, Rank Abundance Curves (RAC), Permutational Multivariate Analysis of Variance (PERMANOVA) and Non-metric Multidimensional Scaling (NMDS) were performed using R software (R version 4.1.3). The packages BiodiversityR^71^, vegan^72^ and ggplot2^73^ were used to examine differences between treatment in plant community composition and visualize community responses to treatment and year.

Species richness and abundance were calculated for 2018, 2019 and 2020 separately for each community. A Kruskal Wallis test was performed to identify significant differences in richness between treatments by year. The number of species were plotted together with the mean and standard error (per treatment) by year. When the results were significant, a post-hoc Dunn’s test was used with Bonferroni adjustments to reveal the treatments which presented richness differences in each year.

The abundance was plotted using Rank Abundance Curves (RAC) on a logarithmic scale considering the percentage cover in August of 2018, 2019 and 2020. In a rank abundance curve, the x-axis represents the species abundance in order of decreasing abundance and the y-axis the relative abundance. Species evenness is reflected in the slope of the line that fits the graph. A steep gradient indicates low evenness as high-ranking species have much higher abundances than low-ranking species. A shallow gradient indicates high evenness as the abundances of different species are similar^71^. This analysis has previously been used as an indicator of the structure of a multispecies community by detailing species-level community changes^74^.

The Permutational Multivariate Analysis of Variance (PERMANOVA) was used to examine differences among treatments in plant community composition and was implemented using the Adonis function in the vegan package^72^. In order to assess community composition differences with PERMANOVA, Bray–Curtis dissimilarity matrices were calculated from species importance values obtained from the averaged percentage cover of each replicate quadrat. Species importance values represent a measure of how dominant a species is in a given community^75^. Subsequently, PERMANOVA was run, including crossed effects of year, treatment as fixed effects, and samples as random effect (Table 1). PERMANOVA is a non-parametric multivariate test used to compare groups. It has been previously used to identify differences in plant community composition associated with environmental variables^70^. A Non-metric Multi-dimensional Scaling analysis (NMDS) was implemented using the metaMDS function in the vegan package with Bray–Curtis dissimilarity matrix^72^ to visualize the responses of the communities to treatments in each year (Figs. 5, 6 and 7). Finally, indicator species analysis was used to assess the species in association with the treatments by year^76^. For the indicator species analysis, the function multipatt was used in the package indicspecies^77^. This function returns the highest indicator value and the p-value as a result. The species with p < 0.05 were represented in the plot. The NMDS species scores and the species resulted from the indicator species analysis were merged using the function left_join in the package dplyr^78^ and plotted using ggplot2 package^73^. All analyses were performed in RStudio 4.1.3.

## Conclusions

Baltic coastal wetlands support high biodiversity and many ecosystem services and so are important targets for conservation. Based on this mesocosm experiment, it can be concluded that the climate change predictions for 2100 for the Baltic Sea will cause significant shifts in coastal wetland plant species richness and abundance.

The present study revealed different responses of wetland communities to altered salinity and water conditions. The Open Pioneer community was shown to be a very dynamic community over time. On the other hand, the Lower Shore and the Upper Shore communities showed significant changes according to time and treatments.

This information is important when considering the protection and potential management of coastal wetlands regarding the species diversity of fauna and flora, as well as ecosystem services such as grazing by livestock.

## Supplementary Information

Supplementary Information.

## Data Availability

Raw data is available in: https://knb.ecoinformatics.org/view/urn%3Auuid%3Ab3b9eb0a-b14f-49d3-aa8c-9262003b645b.
