# Supplementary material for: Experimental climate change impacts on Baltic coastal wetland plant communities
Source: Sci Rep. 2022 Nov 27;12:20362. doi: 10.1038/s41598-022-24913-z (PMC9701761; doi:10.1038/s41598-022-24913-z)
Supplement: Supplementary file 1 — Supplementary Information. [file 41598_2022_24913_MOESM1_ESM.pdf]

Experimental climate change impacts on Baltic coastal wetland plant communities

T.F.Bergamo[a]*, R.D.Ward[a,b,c], , C.B.Joyce[b], M. Villoslada[a,d], K.Sepp[a]

[a]Institute of Agriculture and Environmental Sciences, Estonian University of Life Sciences, Kreutzwaldi 13 5, EE-51014 Tartu, Estonia
[b]Centre for Aquatic Environments, University of Brighton, Cockcroft Building, Moulsecoomb, Brighton BN2 4GJ, United Kingdom
[c]Escola Integrada de Desenvolvimento e Inovação Acadêmica, Federal University of Ceara, Campus do Pici, CEP 60455-760, Fortaleza, Ceara, Brazil
[d]Department of Geographical and Historical Studies, University of Eastern Finland, P.O. Box 111, 80101, Joensuu, Finland

*Address correspondence to T.F. Bergamo. Email thaisafbergamo@gmail.com

Supplementary material. This supplementary material comprises complementary information about the mesocosm experiment design, list of species and additional data presentation.

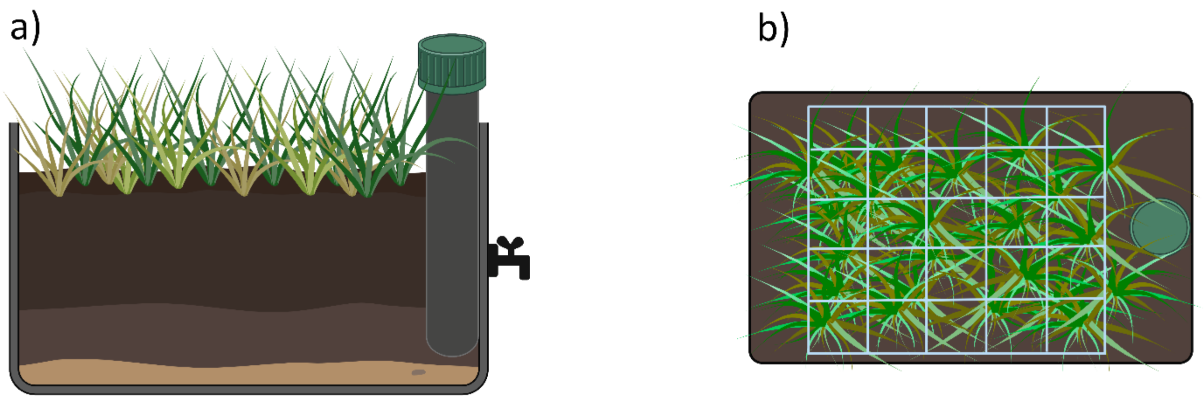

Supplementary Figure S1: Schematic drawing of the experiment design. a) The tap was placed according to the water level of each treatment. Water and salt were added in the tube according to the respective treatment. The tube was kept closed to avoid water evaporation. b) Above view of the sample showing the fixed quadrat divided in 25 sub-quadrats (10x10 cm).

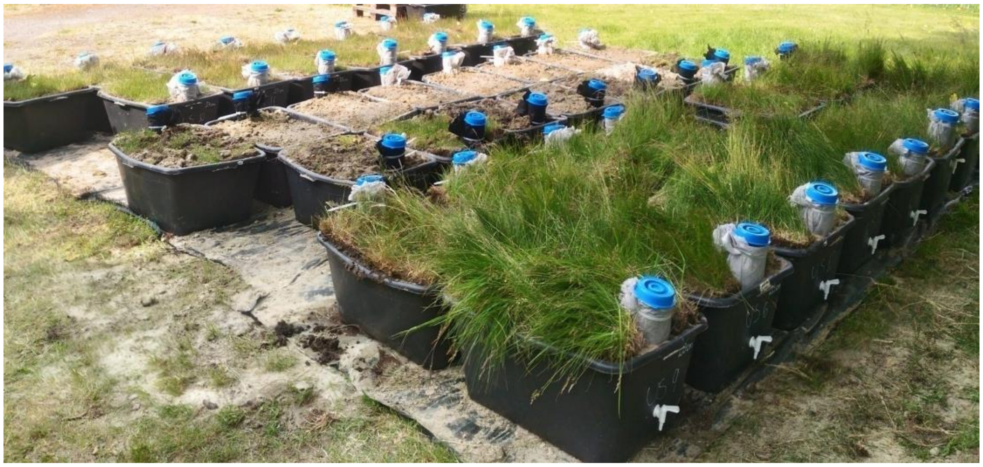

Supplementary Figure S2: Mesocoms experiment in Tartu- Estonia. Photo: Thaisa F. Bergamo.

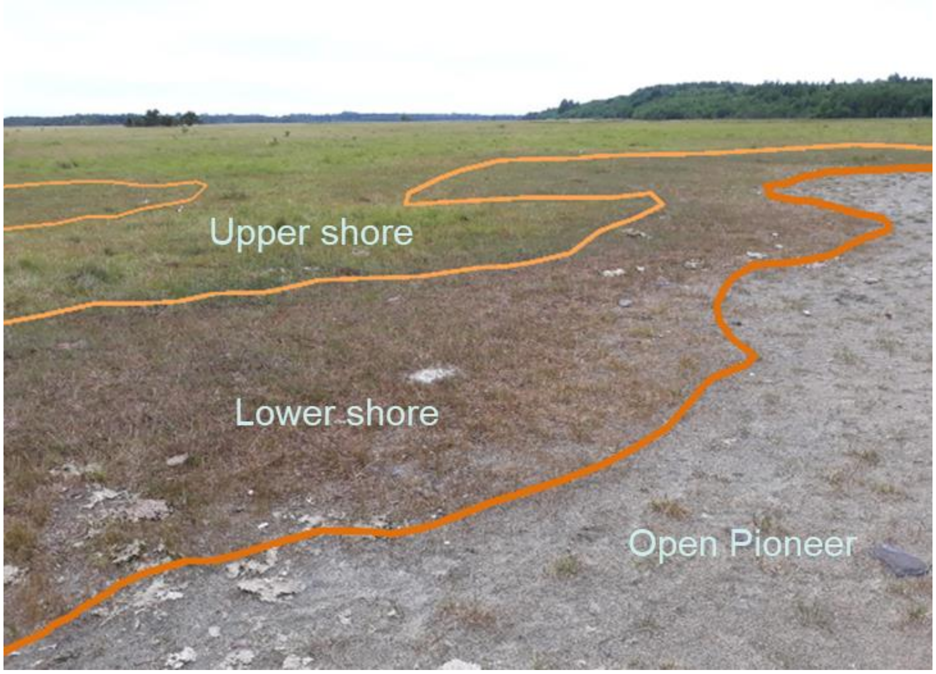

Supplementary Figure S3: The photo shows Tahu North coastal wetland, northwest Estonia (58o58’57.8'' N, 23o34’03.9'' E), and the respective plant communities collected for the experiment. Photo: Chris B. Joyce.

Supplementary Table S1: List of species occurring in the mesocosm experiment in the three plant communities (Open Pioneer, Lower Shore and Upper Shore) during 2018, 2019 and 2020.

	Species / Sampling Year	2018	2019	2020
Open Pioneer	*Atriplex sp*.		x	x
	Agrostis stolonifera	x	x	x
	Carex nigra	x		x
	Centaurium litoralle		x	x
	Eliocharia palustris	x	x	x
	Glaux maritima	x	x	x
	Juncus gerardii	x	x	x
	Moss		x	x
	Odontites versum		x	x
	Plantago maritima	x	x	x
	Puccunellia maritima	x	x	x
	Salicornia europaea	x	x	x
	Spergularia salina	x	x	x
	Suaeda maritima	x	x	
	Trifolium repens		x	x
	Triglochin maritima	x	x	x
Lower Shore	*Agrostis gigantea*	x	x	x
	Agrostis stolonifera	x	x	x
	Blysmus rufus	x	x	x

	Carex flaca	x	x	x
	Carex nigra	x	x	x
	Centaurium litoralle	x	x	x
	Contaurium pulchellum	x	x	
	Cndium dubium	x	x	
	Eliocharis palustris	x	x	x
	Festuca rubra	x	x	x
	Glaux maritima	x	x	x
	Juncus alpinoarticulatus	x		
	Juncus gerardii	x	x	x
	Leontodon autumnalis	x	x	x
	Linum catharticum	x	x	
	Molinea caerula	x	x	x
	Moss	x	x	x
	Odontites versum		x	x
	Plantago maritima	x	x	x
	Poa annua		x	x
	Potentila anseria	x	x	x
	Trifolium pratenses	x	x	x
	Trifolium repens	x		x
	Triglochin maritima	x	x	x
	Triglochin palustris	x	x	x
	Viola canina.		x	
Upper Shore	*Agrostis gigantea*	x	x	x
	Agrostis stolonifera	x	x	x
	Blysmus rufus	x	x	x
	Briza media	x	x	x
	Carex distans		x	x
	Carex flaca	x	x	x
	Carex nigra	x	x	x
	Carex panaciea	x	x	x
	Centaurium litoralle	x	x	x
	Contaurium pulchellum	x	x	x
	Cndium dubium	x	x	x
	Calamagrostis stricta			x
	Comarum palustre			x
	Deschampsia cespitosa	x	x	x
	Eliocharis palustris	x		
	Elymus repens	x	x	x
	Festuca pratensis	x		x
	Festuca rubra	x	x	x
	Filipendula ulmaria	x	x	x
	Galium palustre	x	x	x

Galium uliginosum	x	x	x
Galium verum	x	x	x
Glaux maritima	x	x	x
Juncus africarticulatus	x		
Juncus alpinoarticulatus	x	x	
Juncus gerardii	x	x	x
Lathyrus pratensis	x	x	x
Leontodon autumnalis	x	x	x
Linum catharticum	x	x	x
Lotus corniculatus		x	
Lysimachia vulgaris		x	x
Molinea caerula	x	x	x
Moss		x	x
Plantago maritima	x	x	x
Poa angustifolia	x	x	x
Poa annua	x	x	x
Poa pratensis	x		x
Potentila anseria	x	x	x
Potentilla erecta	x	x	x
Ranunculus acris	x	x	x
Stellaria graminea	x	x	x
Succisa pratensis	x	x	x
Trifolium pratense	x	x	x
Trifolium repens	x	x	x
Triglochin maritima	x	x	x
Triglochin palustris	x		
Vicia cracca	x	x	x
Viola canina.	x		

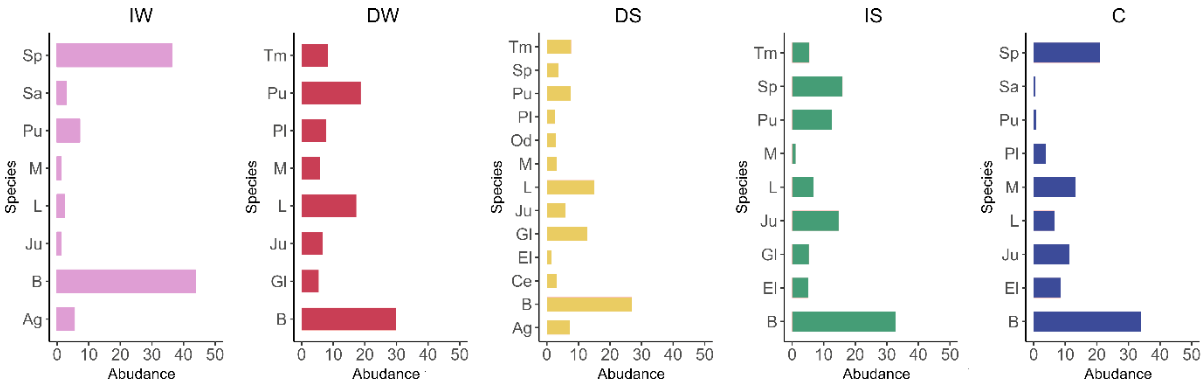

Supplementary Figure S4: Species presence and mean abundance in the Open Pioneer community (in August 2020). Only species with mean abundance exceeding 0.5% in a treatment are shown. Pink: Increased water level

(IW); Red: Decreased water level (DW); Yellow: Decreased salinity (DS); Green: Increased salinity (IS); Blue: Control (C).

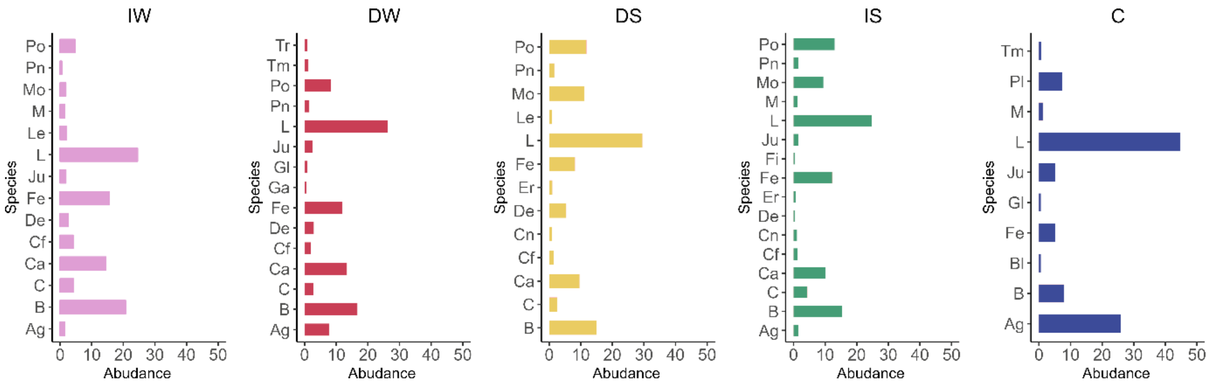

Supplementary Figure S5: Species presence and mean abundance in the Lower Shore community (in August 2020). Only species with mean abundance exceeding 0.5% in a treatment are shown. Pink: Increased water level (IW); Red: Decreased water level (DW); Yellow: Decreased salinity (DS); Green: Increased salinity (IS); Blue: Control (C).

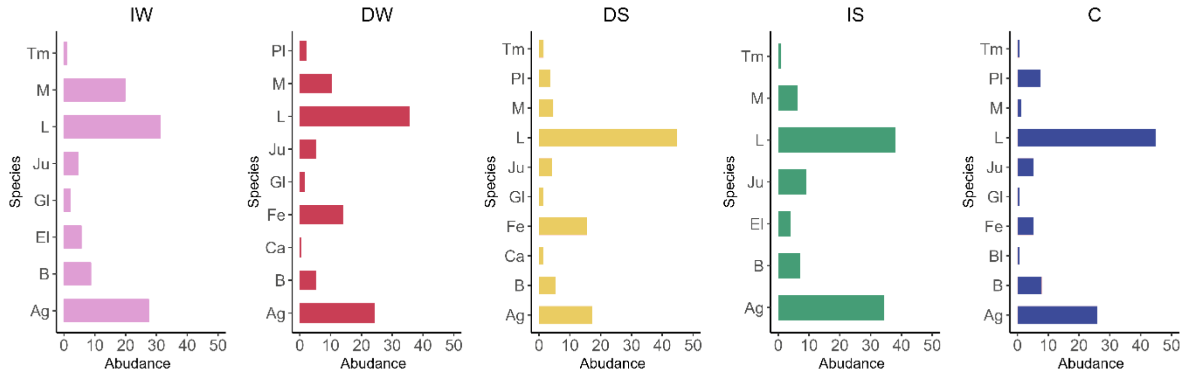

Supplementary Figure S6: Species presence and mean abundance in the Upper Shore community (in August 2020). Only species with mean abundance exceeding 0.5% in a treatment are shown. Pink: Increased water level (IW); Red: Decreased water level (DW); Yellow: Decreased salinity (DS); Green: Increased salinity (IS); Blue: Control (C)
